# Cytokines Associated with Activation of CD4^+^CD25^+^Foxp3^+^ T Regulatory Cells

**DOI:** 10.3390/ijms27042085

**Published:** 2026-02-23

**Authors:** Ranje Al-atiyah, Nirupama D. Verma, Giang T. Tran, Suzanne J. Hodgkinson, Bruce M. Hall

**Affiliations:** 1 Immune Tolerance Laboratory, Ingham Institute, Liverpool, NSW 2170, Australia; ranje.al-atiyah@unsw.edu.au (R.A.-a.); g.tran@unsw.edu.au (G.T.T.); s.hodgkinson@unsw.edu.au (S.J.H.); b.hall@unsw.edu.au (B.M.H.); 2 South West Sydney Clinical Campus, School of Clinical Medicine, Faculty of Medicine and Health, University of New South Wales, Sydney, NSW 2052, Australia; 3 Department of Neurology, Liverpool Hospital, Liverpool, NSW 2170, Australia; 4 Renal Unit, Liverpool Hospital, Liverpool, NSW 2170, Australia

**Keywords:** T regulatory cells, cytokines, tolerance, Th-like Treg

## Abstract

The survival and activation of both effector and regulatory CD4^+^T cells are promoted by cytokines in a complex series of interactions. Alloantigen-specific Regulatory T cells (Treg) constitutively express IL-2 receptor (CD25) and Foxp3. This discovery arose as the cells that transfer the alloantigen-specific transplant tolerance die in culture with specific alloantigens, unless the cultures are supplemented with cytokines from activated lymphocytes. One such cytokine was IL-2, but other cytokines are essential. We describe how the activation of Treg by antigens depends on cytokines produced by antigen-activated effector T cells. These cytokines also drive in parallel the activation of Treg. The Treg are induced to express similar transcription factors and chemokine receptors and have a similar cytokine responsiveness to the activated T effector cells. The activation of Treg by antigens is a two-step process: the first requires cytokines produced by effector T cells early in their activation, and the second step is driven by cytokines produced later by effector T cells during activation. Cytokines from Type 1 responses promote the induction of Th1-like Treg. Likewise, cytokines produced in Type 2, Type 3, and Tfh responses induce different pathways of Treg activation. Understanding the pathways for the activation and expansion of potent antigen-specific Treg will help produce Treg to control allograft rejection or autoimmunity. Currently, the complexity of the numerous potential pathways of activation of Treg remains incompletely understood. The dogma that IL-2 is the only driver of Treg activation may have hindered the development of highly potent antigen-specific Treg for therapy.

## 1. Introduction

The survival of lymphocytes, with or without antigen activation, requires cytokines. Early studies of lymphocytes found they die in culture, leading to the erroneous conclusion that lymphocytes have no functional role [1]. The survival of naïve lymphocytes, which express the IL-7 receptor (IL-7R) (CD127), depends upon cytokines such as IL-7 [2]. Cytokines are key to the survival, activation, and function of all CD4^+^ T cells. This includes CD4^+^CD25^+^Foxp3^+^ T regulatory cells (Treg) that are CD127^lo^, and depend upon IL-2 binding to CD25 to promote their survival and expansion [3]. Cytokines are produced by innate immune cells such as innate lymphoid cells (ILCs), monocytes/macrophages, natural killer (NK) cells, dendritic cells (DCs), and somatic cells, as well as by antigen-activated T cells and B cells [4].

The generation, maintenance, and execution of T-cell-mediated immune responses depend upon the recognition of specific antigens by T-cell receptors [5,6]. The full activation of lymphocytes requires cytokines binding to specific cytokine receptors expressed by the activated lymphocytes. These cytokines can promote the proliferation, migration, and effector function of lymphocytes. Depending on the nature of the antigen, effector CD4^+^ T cells with different functions are activated, including T helper (Th) 1, Th2, Th17, and T follicular helper (Tfh) cells. The pathways for the generation of these distinct effector T cells are driven by different cytokines, which are well-described. In contrast, the pathways for the full activation of antigen-specific Treg are less well-defined.

The cytokines produced by activated effector CD4^+^ T cells act on the CD4^+^ T cells themselves as well as on other cell types, including CD8^+^ T cells, B cells, monocytes/macrophages, and somatic cells in the inflamed tissue. In addition, these cytokines promote the activation of Treg. This review will focus on how cytokines produced as part of effector T-cell activation act on antigen-activated Treg to promote their survival, activation, and migration to inhibit the effector responses.

As background, we describe our work that identified alloantigen-specific CD4^+^CD25^+^ Treg [7,8,9]. These CD4^+^ Treg were shown to need specific antigen stimulation and cytokines from activated lymphocytes to survive. The cytokines that promote antigen-specific Treg activation are not yet fully defined. Here, we challenge the common belief that IL-2 is central to all Treg function and describe a key role for other inflammatory cytokines in activating Treg.

In 2025, the Nobel Prize in Medicine or Physiology recognised the importance of T regulatory cells, which were CD4^+^ and expressed the IL-2Rα. These CD4^+^CD25^+^ T cells were first described to mediate allograft tolerance, and, later, Sakaguchi showed CD4^+^CD25^+^ T cells prevent autoimmunity. Brunkow and Ramsdale were recognised for identifying Foxp3, a transcription factor that confers regulatory function on T cells and regulates cytokine gene expression. Other transcription factors drive the induction and function of other lymphocyte subsets, inducing different cytokines and cytokine receptors. The full activation of potent Treg induces effector-T-cell transcription factors, such as T-bet in Foxp3-expressing Treg. The effector-T-cell transcription factor induces the expression of inflammatory cytokines such as IFN-γ.

This review presents our perspective on the critical role cytokines play in the activation and control of T regulatory cells, which derives from our observation that antigen-specific Treg die without stimulation by a variety of cytokines, one of which is IL-2 acting on the CD25 on Treg.

### 1.1. Lineages of Effector CD4^+^ T Cells—Th1/Th2/Th3/Th17/Tfh

The activation of naïve T cells by antigens, including autoantigens, infection, tumours, or alloantigens, requires cytokines to promote the proliferation, migration, and effector function of these cells. With Th cells, depending on the nature of the antigen, this activation leads to the generation of functionally different effector cells, including Th1, Th2, Th17, and Tfh cells. The pathways of generation of these distinct effector cells differ and involve different cytokines. As effector T cells are activated, they are induced to express transcription factors that promote the induction of cytokines, chemokine receptors that programme their migration to sites of inflammation and effector molecules such as granzymes and perforin. These pathways are well-defined [10].

### 1.2. Th1 and Th2 Responses

In 1986, Mosmann et al. [11] described how mouse CD4^+^ Th cells can differentiate into Th1 and Th2 types. Later, this Th1/Th2 paradigm was identified in human Th cells [12]. The various Th cells differ in the cytokines they produce and in the inflammatory responses they induce. Th1 cytokines include IL-2, IFN-γ, Lymphotoxin, IL-12, and IL-18, and are considered pro-inflammatory. Th2 cytokines include IL-4, IL-5, IL-10, IL-13, and IL-33, and are considered anti-inflammatory and thought to suppress the immune response [13,14,15,16]. There is crosstalk between Th1 and Th2, with Th2 cytokines IL-4 and IL-10 inhibiting Th1 cells, while the Th1 cytokine IFN-γ inhibits Th2. It was postulated that Th2 responses may be tolerance-inducing. Later, Th3 cells producing TGF-β were described. Some cytokines are produced by both Th1 and Th2. These include IL-3, tumour necrosis factor alpha (TNF-α), IL-6, and granulocyte-macrophage colony-stimulating factor (GM-CSF). Th1-associated responses are now called Type-1; Th2 are Type-2, and Type3 is an IL-17-associated immune response.

### 1.3. From CD8^+^I-J^+^ Suppressor Cells to CD4^+^CD25^+^ T Cells

**Figure 1:**
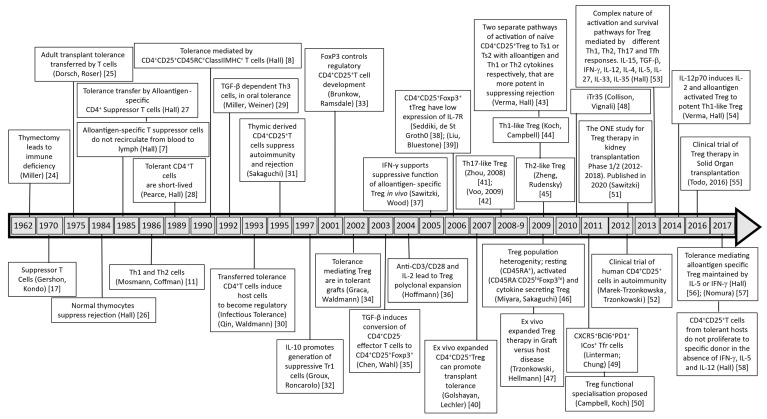
Timeline related to T regulatory cells and cytokines [7,8,11,17,24,25,26,27,28,29,30,31,32,33,34,35,36,37,38,39,40,41,42,43,44,45,46,47,48,49,50,51,52,53,54,55,56,57,58].

Suppressor T cells, first described in the early 1970s [17], were identified as CD8^+^ T cells expressing the I-J region of MHC [18,19]. When the characterisation of genes in the mouse MHC class II region did not identify the I-J locus [20], the concept of suppressor cells was almost totally vanquished by mainstream immunology [21]. At this time, in the 1980s, we identified in a murine transplant tolerance model that CD4^+^ T cells, not CD8^+^ T cells, could transfer tolerance and suppress rejection [22,23]. Since then, the Treg field has substantially progressed with a better characterisation of the Treg phenotype, function, and growth requirements (Figure 1). The role of cytokines in the induction and survival of these alloantigen-activated CD4^+^ T cells is the focus of this review.

## 2. CD4^+^CD25^+^ T Cells Mediate Transplant Tolerance

Our studies found that, in some MHC-incompatible strain combinations, the temporary inhibition of the rejection response in adult animals leads to long-term cardiac allograft survival without ongoing immunosuppression. Animals with long-surviving (>100 days) fully allogeneic heart transplants lose the capacity to reject a second skin graft from the same donor strain, but not a third-party graft [59]. These animals are operationally tolerant (OT). This tolerance is alloantigen-specific but takes time to develop [27,60].

**Figure 2:**
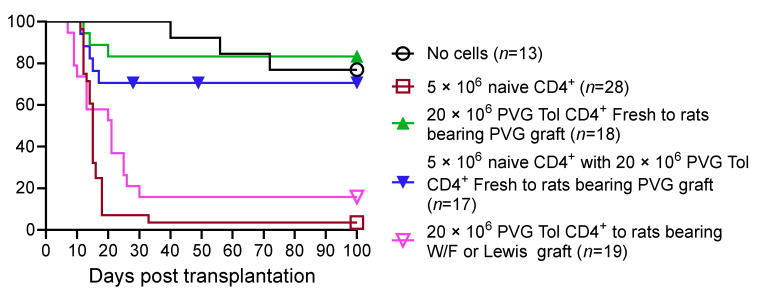
Naïve T cells restore rejection in irradiated rats while tolerant (Tol) CD4^+^ cells transfer tolerance to specific donor-strain graft from PVG rats, but not third-party graft from Wistar-Furth (W/F) or Lewis rats.

We studied peripheral lymphocytes (lymph node and spleen cells) from these OT hosts (Tolerant cells) and compared their function to that of lymphocytes from naïve hosts (Naïve cells). Tolerant cells were adoptively transferred to hosts depleted of lymphocytes by whole-body irradiation. The irradiated adoptive hosts do not reject their cardiac allograft [7] (Figure 2; based on our published data [8,56,57,61,62]). In contrast, the lymph node and spleen cells or CD4^+^ T cells from naïve animals restore rejection in nearly all hosts [27]. CD8^+^ T cells alone are ineffective in restoring rejection [63].

Transferring lymphoid cells or CD4^+^ T cells from OT rats does not lead to the rejection of specific donor cardiac allografts, but third-party MHC-incompatible cardiac allografts are rejected [22,23]. These freshly isolated tolerant cells also suppress the rejection mediated by naïve CD4^+^ cells. The transfer of naïve lymphoid cells to OT hosts does not affect rejection. CD8^+^ T cells, B cells, or antibodies from OT hosts do not transfer tolerance. This was one of the first reports that CD4^+^ T cells, not CD8^+^ T cells, mediate tolerance [27].

These OT hosts have peripheral lymphocytes that respond ex vivo to specific donor alloantigens, however. CD4^+^ T cells from OT hosts respond like naïve lymphocytes in mixed lymphocyte cultures [28] and in graft-versus-host assays [64]. Thus, the OT hosts are not clonally depleted of donor-reactive lymphocytes. Therefore, the mechanism of tolerance is not clonal deletion, as described in detail [65].

Further, CD4^+^ T cells from OT hosts suppress the rejection mediated by co-transferred naïve CD4^+^ T cells. Larger numbers of cells from OT than naïve cells are required. We use a ratio of 4:1 to naive CD4^+^ T cells. These CD4^+^ T cells from OT hosts do not suppress third-party allograft rejection, demonstrating their suppression is alloantigen-specific [27].

In hosts induced to not reject their graft, who are developing OT, the ability to accept a second donor-specific graft takes >100 days to develop. The tolerance-transferring cells take time to dominate the host response to the allograft. The suppression of rejection by CD4^+^ T cells from hosts treated to induce OT is only evident >100 days post-transplantation. Thus, OT in adult rodents takes some time to develop and includes an induction period where CD4^+^ T cells cannot transfer tolerance as they mediate rejection.

**Figure 3:**
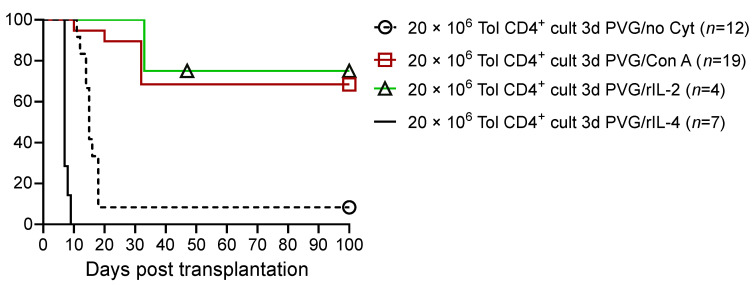
Effect of cytokines (Cyt) on ability of tolerant cells to transfer tolerance to specific donor (PVG). Tolerant (Tol) CD4^+^ cells lose the capacity to transfer tolerance upon culture (cult) without cytokines.

The in vitro culture of these CD4^+^ T cells from OT hosts for 3 days in mixed lymphocyte culture (MLC) leads to a loss of their ability to transfer tolerance (Figure 3; based on our published data [9,56,57,62]). IL-2 supports the survival of tolerant CD4^+^ cells, while IL-4 does not. A culture with no stimulator cells, with donor-specific alloantigen stimulator cells or idiotype-expressing alloantigen-activated T cells, does not promote the survival of tolerance-transferring CD4^+^ T cells [9]. This suggests that the regulatory CD4^+^ T cells are either reactivated to effector CD4^+^ cells or die in culture.

These observations led us to explore if cytokines produced by activated lymphocytes could promote the survival of tolerance-transferring CD4^+^ T cells in culture [9,66]. We found that a mixture of cytokines generated as a supernatant of Concanavalin A-stimulated lymphocytes (Con A s/n), together with specific donor alloantigens, promotes the survival of tolerance-transferring CD4^+^ T cells [9]. A culture with Con A s/n in the absence of donor alloantigens does not maintain the Treg function of CD4^+^ T cells, nor does stimulation with idiotype-expressing cells combined with Con A s/n [9].

These findings raised the question as to which cytokine/s in Con A s/n could sustain the tolerance-mediating CD4^+^ Treg. Con A polyclonally activates T cells but not B cells. At that time, Con A s/n was considered a source of the activated T-cell cytokine IL-2. Con A s/n contains many other cytokines, including IFN-γ, IL-4, and IL-10 [67].

We then examined whether the tolerance-transferring CD4^+^ T cells from OT hosts express the IL-2 receptor CD25. The depletion of CD25^+^ cells removes tolerant CD4^+^ T cells’ capacity to transfer tolerance [8]. This was the first description of a CD4^+^CD25^+^ Treg and a potential role of IL-2 in promoting Treg.

At that time, IL-2 was considered a pro-inflammatory cytokine that promoted the activation of effector T cells, especially in the rejection response [68,69]. Therapy with monoclonal antibody (mAb) to CD25 was used to prevent rejection and is still used in renal transplant patients [70,71].

**Figure 4:**
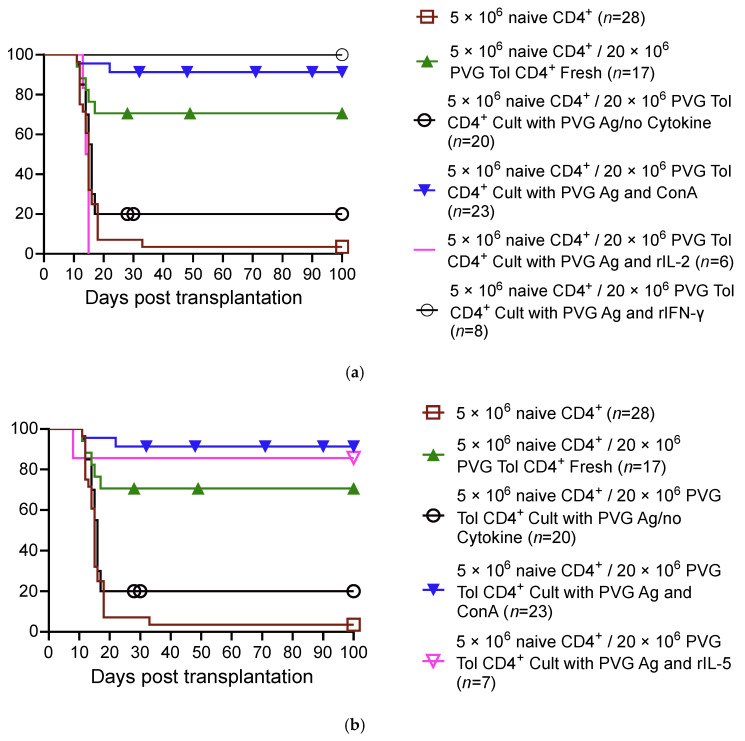
Ability of tolerant (Tol) CD4^+^ cells cultured (Cult) with specific donor alloantigen (PVG) and Th1 (**a**) or Th2 (**b**) cytokines to suppress rejection of the specific donor (PVG) graft mediated by naïve CD4 cells.

We showed that CD4^+^ T cells from OT hosts, after culture with IL-2 and specific donor alloantigens, transfer tolerance [9]. However, CD4^+^ T cells from OT hosts that are cultured with specific donor alloantigens and IL-2 on transfer do not suppress the rejection mediated by co-transferred naïve CD4^+^ T cells [9]. IL-2’s failure in preserving the suppressive capacity/promoting the survival of tolerance-transferring CD4^+^ T cells demonstrated a potential role for other cytokines present in Con A s/n [9]. This led us to examine the role of other cytokines including Th1 (Figure 4a; based on our published data [9,57]) and Th2 (Figure 4b; based on our published data [56]) cytokines in maintaining the suppressive function of alloantigen-specific CD4^+^CD25^+^ Treg from tolerant animals (control data in Figure 4a,b based on our published data) [8,9,56,57,62]. Late Th1 cytokine IFN-γ (Figure 4a) and late Th2 cytokine IL-5 (Figure 4b) suppress the rejection mediated by naïve CD4 cells.

We also identified that these tolerance-transferring CD4^+^ T cells express other markers, which include MHC-class II and CD45RC, characterising these cells as CD4^+^CD25^+^CD45RC^+^MHCII^+^ cells [8]. MHC-class II expression and the lack of CD45RA are now established markers of activated CD4^+^CD25^+^ Treg. CD45RC is expressed by activated T cells, whereas naïve/resting T cells express CD45RA. Apart from CD45RC, activated/memory T cells also express CD45RO and CD45RB, while lacking CD45RA expression.

Five years after this first description of CD4^+^CD25^+^ Treg, this phenotype of peripheral lymphoid cells was reported to prevent autoimmunity in a day 3–5 neonatal thymectomy model by Sakaguchi et al. [31]. These CD4^+^CD25^+^ T cells are thymus-derived naïve/resting Treg, which are not antigen-specific. While these cells express the IL-2 receptor, CD25, highlighting its importance in Treg development and survival, IL-2 promotes their polyclonal expansion. Naïve/resting Treg have a lower suppression potency than the activated Treg described in transplant tolerant rats. It is the latter, the activated Treg, that restore tolerance in an antigen-specific manner. Both naïve/resting Treg and activated antigen-specific Treg express Foxp3, the Treg transcription factor [72]. Foxp3 inhibits the Treg expression of IL-2 but induces CD25 expression.

CD25 is the IL-2Rα, and the survival and expansion of naïve/resting CD4^+^CD25^+^Foxp3^+^ Treg is dependent on the T-cell-derived cytokine IL-2. Specific-antigen-activated Treg, however, are promoted by other cytokines from activated T cells in addition to their requirement of specific antigen stimulation.

CD4^+^CD25^+^Foxp3^+^ Treg are a minor population of CD4^+^ T cells (<10%). Within this minority, the activated-antigen-specific Treg are only a small fraction of cells.

There are also CD8^+^ Treg and regulatory B cells; however, this review focuses on CD4^+^ Treg.

Our group’s focus has been to study the cytokine requirements of these antigen-specific Treg when activated in response to alloantigens or an autoantigen [65,73,74].

## 3. Cytokines Associated with Naïve/Resting CD4^+^CD25^+^Foxp3^+^ Treg

Naïve/resting CD4^+^CD25^+^Foxp3^+^ Treg inhibit antigen-presenting cells by blocking and downregulating the co-stimulatory molecules CD80 and CD86 which bind to CD28 on T cells and provide a signal to activate T cells [75]. This regulatory function helps control autoimmunity [76]. Naïve Treg can also mediate suppression by the release of TGF-β and/or IL-10 [77,78,79,80]. CTLA4 expressed by Treg and activated effector T cells blocks the CD28 ligands [81].

The activation of CD3 and CD28 by mAb combined with high levels of IL-2, in the absence of an antigen, is required to induce the proliferation and polyclonal expansion of naïve CD4^+^CD25^+^Foxp3^+^ Treg [82]. The maintenance and expansion of these Treg in vivo and in vitro require IL-2.

**Interleukin 2.** The main function of IL-2 is centred on the activation of effector lineage T cells and CD4^+^CD25^+^Foxp3^+^ T cells. Activated effector lineage T cells are induced to express and secrete IL-2, which acts in both an autocrine and paracrine manner on T cells.

IL-2 assists in the proliferation, differentiation, and activation of both pro-inflammatory and anti-inflammatory cells [83,84] and was originally known as T-cell growth factor due to its support of T-cell survival in vitro [85,86,87]. This cytokine is essential in the early stages of naïve-T-cell priming, promoting the differentiation of naïve T cells into Th1 and Th2 cells, supporting Th9 cell and Treg differentiation, while inhibiting Th17 and Tfh cell differentiation.

There are three IL-2 receptor chains; IL-2Rα (CD25), IL-2Rβ (CD122), and IL-2Rγ (CD132). They can form complexes of varying affinities. The trimeric complex has the highest affinity for IL-2. The effect of IL-2 is mediated differently on naïve T cells and Treg depending on the IL-2R subunits expressed.

Treg express trimeric IL-2 receptor. IL-2Rα is constitutively expressed by Treg at high levels, but will bind IL-2 with low affinity [88]. Treg do not produce IL-2 and are dependent on exogenous IL-2 [89].

The dimeric receptor, with IL-2Rβ and IL-2Rγ, is expressed on resting T cells and binds IL-2 with intermediate affinity. During activation by specific antigens, these CD4^+^CD25^−^Foxp3^−^ T cells express IL-2Rα. These activated Foxp3^−^ T cells become CD4^+^CD25^+^ T cells and are the main effectors of rejection.

Whether IL-2 will have a pro-inflammatory or anti-inflammatory effect can be dictated by which receptors are available to bind the cytokine. This is demonstrated in studies with anti-IL-2 antibodies. Antibody JES6-1 promotes the formation of the IL-2Rα, IL-2Rβ, and IL-2Rγ complex, which, in turn, supports Treg development, while the addition of S4B6 antibody blocks the IL-2:IL-2Rα interaction, allowing the formation of only the IL-2Rβ and IL-2Rγ complex, which promotes T effector cell growth [90]. IL-2 is responsible for maintaining the gene expression necessary for regulating the growth and metabolism of the scells [3].

In vitro studies examining CD4^+^CD25^+^ T cell activation by alloantigen show naïve CD4^+^CD25^+^ T cells alone have low levels of proliferation, which is enhanced by the addition of IL-2. Proliferation with IL-2 occurs with responses to alloantigens or self-antigens. This is the first step in the activation of naïve/resting Treg towards the generation of antigen-specific Treg.

IL-2 has been shown to be indispensable for the maintenance of a tolerogenic state in mice. IL-2 knockout mice lack regulatory functions and develop a lymphoproliferative disorder confirming IL-2’s role in self-tolerance and in preventing autoimmunity [91,92,93].

**Interleukin-7.** IL-7 is the cytokine required for the survival of effector lineage CD4^+^ T cells [94]. Being a haemopoietic growth factor, IL-7, is produced by stromal cells in thymus, bone marrow, as well as follicular dendritic cells [95,96,97]. IL-7 is also produced by somatic cells, such as epithelial cells, keratinocytes, hepatocytes, and neurons [98,99,100,101]. The production of IL-7 is mainly constitutive but can be enhanced by cytokines such as IFN-γ [102]. Normal lymphocytes do not produce IL-7 [103].

IL-7 promotes the development, survival, and function of activated lymphocytes, T cells (memory and activated), B cells, NK cells, and dendritic cells. In addition, IL-7 activates the JAK-STAT, PI3K-Akt, and NAPK pathways [104]. The rearrangement and expression of T-cell receptor genes and immunoglobulin genes is facilitated by IL-7.

IL-7R is a heterodimer of IL-7Rα (CD127) and the common γ chain (IL-2Rγ) (CD132). IL-7Rα combines with CD132 (common gamma chain), which is shared with cytokines like IL-2, to make the functional heterodimeric IL-7 receptor. Naïve effector lineage T cells express IL-7Rα and need IL-7 to promote their survival [38]. Defects in IL-7 and IL-7Rα result in a severe combined immune-deficiency (SCID), with a lack of T cells [105].

Human CD4^+^CD25^+^Foxp3^+^ Treg have a low or no expression of IL-7Rα [104]. IL-7 is not required for the survival of CD4^+^CD25^+^Foxp3^+^ Treg cells. The low expression of CD127 is used to identify CD4^+^CD25^+^Foxp3^+^ Treg and separate them from effector lineage CD4^+^ T cells [38,39].

## 4. Examination of the Role of Cytokines, Other than IL-2, in Activation and Survival of Antigen-Activated CD4^+^CD25^+^Foxp3^+^ Treg

**Figure 5:**
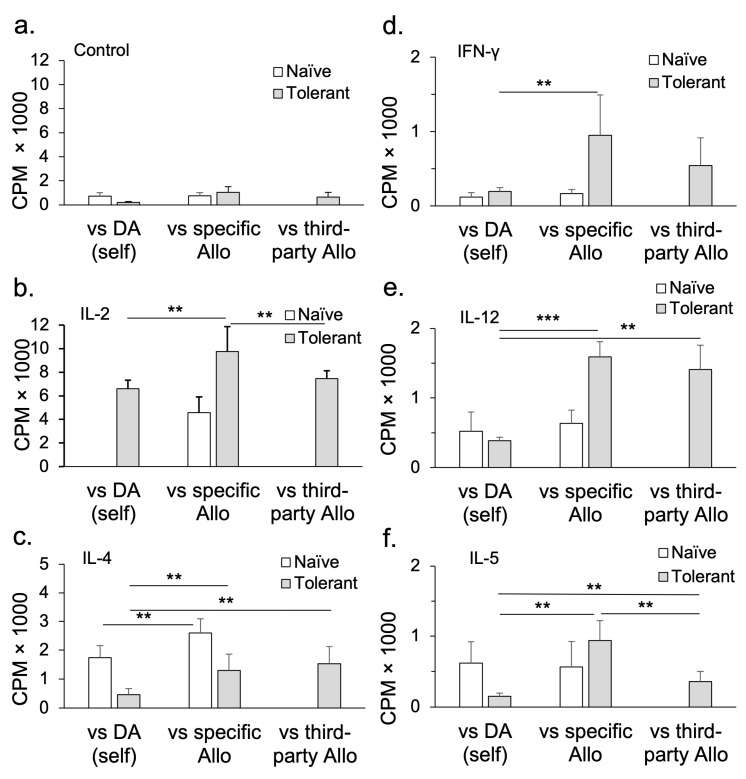
In vitro proliferation of CD4^+^CD25^+^ cells from naïve and tolerant DA rats in the presence of Th1 ((**b**) IL-2, (**d**) IFN-γ and (**e**) IL-12) or Th2 ((**c**) IL-4 and (**f**) IL-5) cytokines and antigen. For tolerant cells, antigen is either specific (same as donor antigen) or third-party. Cytokines were added as culture supernatant from cytokine gene transfected CHO cells. (**a**) Control refers to supernatant from non-transfected CHO cells. Significance is indicated by ** *p* < 0.01 and *** *p* < 0.001.

We examined the effects of other T-cell-derived cytokines on CD4^+^CD25^+^ T cells [43,54,56,57,58,62,106,107]. Naïve/resting CD4^+^CD25^+^ T cells, upon activation with an alloantigen or autoantigen and rIL-2 or rIL-4, have a small proliferative response to self and allogeneic stimulator cells. This response is enhanced by either rIL-2 or rIL-4, but not by rIFN-γ, rIL-12, rIL-5, rIL-10, rIL-13, or rTGF-β [43] (Figure 5; based on our published data [56,58]).

In contrast, the culture of CD4^+^CD25^+^ T cells from animals with OT to an allograft does not proliferate to specific donors in cultures lacking cytokines, but responds like naïve CD4^+^CD25^+^ T cells to third-party allogeneic stimulation. This is consistent with the antigen-specific CD4^+^CD25^+^ T cells dying as they lack key cytokine stimulation. We found rIL-2 and rIL-4 have a polyclonal effect on these activated Treg (Figure 5). Other cytokines, including rIFN-γ, rIL-12, and rIL-5, promote the proliferation of CD4^+^CD25^+^ T cells from OT hosts to specific donors but not third-party or self. These cytokines are produced after IL-2 in an immune response to antigen. Many cytokines, including rIL-10, rIL-13, and rTGF-β, had no effect on CD4^+^CD25^+^ T cells from OT hosts [58].

These studies suggest activated CD4^+^CD25^+^ Treg from hosts with tolerance to an allograft may need cytokines other than IL-2 or IL-4.

This led us to hypothesise that the activation pathways of CD4^+^CD25^+^Foxp3^+^ Treg may depend upon the cytokines produced by activated effector CD4^+^ T cells. We demonstrated that the initial culture of CD4^+^CD25^+^ cells for 3–4 days with antigens and Th1 (IL-2) or Th2 (IL-4) cytokines induces the expression of receptors for cytokines produced late in the respective effector response, leading to the differentiation of Treg down two separate pathways, and also enhances the suppressive potency of Treg [43]. We also proposed that the late cytokines produced during specific immune responses would further activate these antigen-specific Treg to the respective Th-like Treg, defining the full activation pathway as a two-step process.

Our studies on CD4^+^ cells from OT animals support our proposed model that tolerant cells express the receptors for late-phase Th1 or Th2 cytokines like IL-5, IFN-γ, or IL-12, in contrast to naïve Treg that do not express these. Freshly isolated tolerant CD4^+^CD25^+^ cells express mRNA for *Il5ra* and *Ifngr* [56], but not for *Gata3* or *T-bet* [56]. The culture of these tolerant cells with specific antigens and rIL-5 [56] or rIFN-γ [57] maintains the expression of *Il5ra* or *Ifngr*, respectively, and promote their in vitro proliferation.

Further, the culture of these tolerant CD4^+^ T cells with specific donor stimulator cells and either rIFN-γ or rIL-12 maintain *Ifngr* [57] or *Il-12rb2* [58] expression on these cells, and IFN-γ [57] also preserves their tolerance transferring ability. Similarly, tolerant cells, when cultured with specific donor stimulator cells and rIL-5, but not with rIL-4 or no cytokine, retain *Il5ra* expression and support their in vitro proliferation and tolerance-transferring ability [56]. In contrast, the culture of tolerant CD4^+^ T cells with rIL-4 [62] or rIL-2 [9] alone or with specific donor stimulator cells alone does not sustain the tolerance-mediating cells and they acquire the capacity to mediate rejection. Taken together, these results suggest that tolerance-mediating CD4^+^ T cells become dependent on cytokines expressed late in the immune response, such as IFN-γ, IL-12, or IL-5, and no longer depend on cytokines produced at the beginning of an effector T cell response, such as IL-2 or IL-4.

Further support for a role for late cytokines in Treg activation and survival is that therapy with IL-5 [108] or IL-12 [109,110] delays graft rejection and promotes Treg.

**Figure 6:**
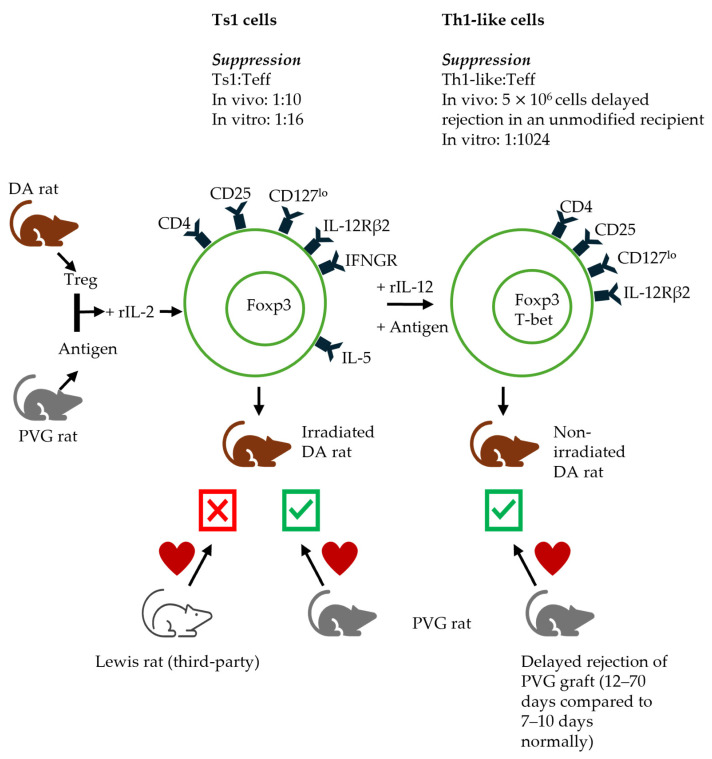
Activation of naïve Treg with rIL-2 and alloantigen (Ts1 Pathway). Culturing CD4^+^CD25^+^ Treg from naïve DA rat with antigen from a PVG rat and rIL-2 leads to the generation of more potent Treg (Ts1 cells) that are CD4^+^CD25^+^CD127^lo^Foxp3^+^IFNGR^+^IL12Rβ2^+^. These Ts1 cells suppress PVG heart allograft rejection in vivo at a ratio of 1:10 to naïve CD4^+^ cells in irradiated DA rats but fail to suppress rejection of a heart from third-party (Lewis) allograft. In vitro, Ts1 cells suppress naïve CD4^+^ T cells’ proliferation at a ratio of 1:16 (Ts1: Teff), as compared to a ratio of 1:1 when using naive/resting Treg. Further stimulation of Ts1 cells with antigen from a PVG rat and rIL-12 results in development of Th1-like Treg that suppress at a ratio of 1:1024 (Th1-like Treg: Teff). Furthermore, 5 × 10^6^ Th1-like Treg delay rejection of a PVG heart graft in a non-irradiated DA rat, whereas normal rejection time is 7–10 days. The enclosed tick refers to suppressed/delayed graft rejection, while enclosed x indicates graft rejection was not suppressed.

**Figure 7:**
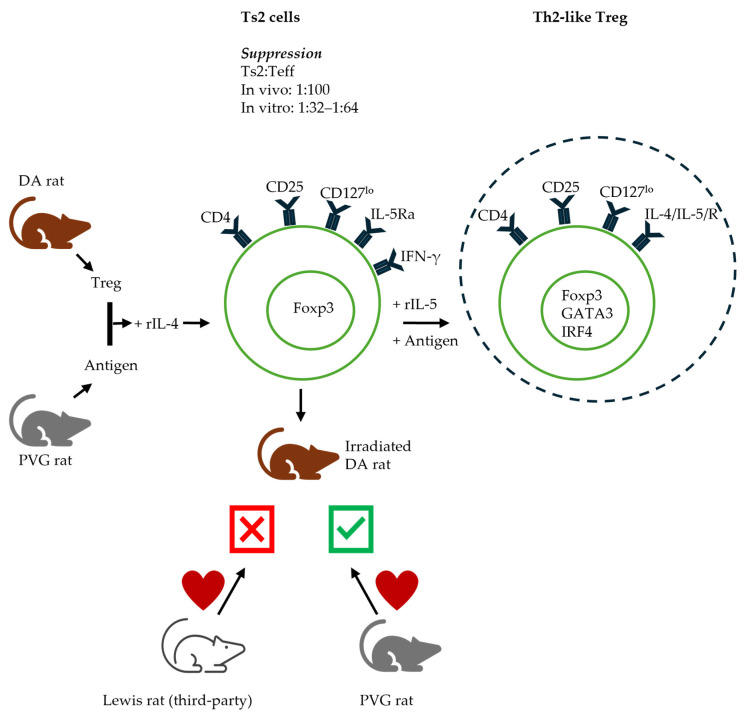
Activation of naïve Treg with rIL-4 and alloantigen (Ts2 Pathway). Culture of CD4^+^CD25^+^ Treg from naïve DA rat with irradiated PVG cells and rIL-4 leads to the generation of more potent Treg (Ts2 cells) that are CD4^+^CD25^+^CD127^lo^Foxp3^+^IL-5Rα^+^. These Ts2 cells suppress PVG heart allograft rejection in vivo at a ratio of 1:10 to naïve CD4^+^ cells in irradiated DA rats but not third-party (Lewis) allograft. Ts2 cells suppress naïve CD4^+^ T cells at a ratio of 1:32–1:64 (Ts2:Teff) in vitro, as compared to a ratio of 1:1 with naïve Treg. In vitro stimulation of Ts2 cells with PVG antigen and rIL-5 results in development of Th2-like Treg (cell in dotted line circle) that exhibit similar phenotype of Th2 cells, including expression of the transcription factors GATA3 and IRF4, as well as expression of IL-4 and IL-5 and their respective receptors. The enclosed tick refers to suppressed/delayed graft rejection, while enclosed x indicates graft rejection was not suppressed.

The demonstration of the Ts1 and Ts2 pathways (Figure 6 and Figure 7 respectively) is consistent with the distinct separate pathways for the activation of naïve/resting CD4^+^CD25^+^Foxp3^+^ T cells by either Th1 (IL-2) or Th2 (IL-4) cytokines [43]. These proposed pathways are described in more detail in the respective section of Th responses.

## 5. Type 1 Immune Responses: Effect of Th1 Cytokines on Treg Function

**Ts1 cells.** In our proposed Ts1 pathway, naïve/resting CD4^+^CD25^+^ Treg cultured with alloantigens and rIL-2 induce Treg that express cytokine receptors for late Th1 cytokines, specifically receptors for IL-12 (IL-12Rβ2) and IFN-γ (IFNGR) but not receptors for late Th2 cytokines like IL-5 [43]. These cells are named Ts1 and express Foxp3 and *Ifngr* and *Il12rb2* mRNA, while *Ifnγ* expression is reduced [43]. In vitro, Ts1 Treg are able to suppress naïve CD4^+^ T cells at a ratio of 1:32 to 1:64 (Ts1: effector T cells), as compared to a ratio of 1:1–1:2 with unstimulated naïve/resting Treg [111]. Ts1 Treg suppress PVG heart allograft rejection in irradiated rats restored with 5 × 10^6^ naïve CD4^+^ T cells at a ratio of 1:10 in DA rats. Ts1 cells reject a third-party fully allogeneic Lewis graft. This demonstrates the enhanced suppressive ability of Ts1 Treg and antigen specificity [43]. Suppression by naïve CD4^+^CD25^+^ Treg is not antigen-specific [43,57].

Ts1 cells were also studied in two models of autoimmunity, EAE and EAN. CD4^+^CD25^+^Foxp3^+^ T cells from naïve Lewis rats are activated in vitro with rIL-2 and specific autoantigen MBP for the EAE model and PNM for EAN. After three days in culture, these Treg express mRNA for *Ifngr* and *Il12rb2*, but no *Tbet*, *Il2*, or *Ifnγ*. These specific autoantigen-activated Treg inhibit EAN and EAE in Lewis rats [107]. Ts1 cells generated to a third-party autoantigen (Renal Tubular antigen) do not inhibit paralysis in both EAE [112] and EAN [107], demonstrating the antigen specificity of these cells in vivo. On the other hand, fresh CD4^+^CD25^+^Foxp3^+^ T cells from naïve Lewis rats do not inhibit either EAE or EAN. This demonstrates the requirement of the cytokine-driven activation of antigen-specific Treg.

These cultures induce phenotypic changes, with a significant proportion of cells expressing CD8α and CD8β, as well as CD4. The depletion of the CD8^+^ cells removes the capacity of the cultured cells to suppress EAE [112]. The induced CD8 expression may be a useful marker of the antigen-activated CD4^+^CD25^+^ Treg. Similarly, the dual positive CD4^+^CD8^+^CD25^+^Foxp3^+^ T cells mediate the acceptance of allografts [113]. The cultured cells have an increased expression of CD62L, suggesting they migrate to secondary lymphoid tissues, not to sites of inflammation in non-lymphoid tissues.

**Th1-like Treg.** The further alloactivation of Ts1 Treg with specific antigens (antigens used in the first step of activation of naïve Treg, PVG) and rIL-12 for 3 days results in the production of Treg (Th1-like) expressing T-bet, the Th1 transcription factor, as well as Foxp3 and IFN-γ [54]. Th1-like Treg suppress naïve CD4^+^ T cell proliferation at a ratio of 1:1024 to effector cells compared to Ts1, which suppress at a ratio of 1:32–64. Furthermore, 5 × 10^6^ of these Th1-like Treg delay PVG allograft rejection in unmodified DA hosts, demonstrating their enhanced suppressive capacity. These Th1-like Treg were described by Koch in Th1-mediated inflammation [44] and by Dominguez-Villar in autoimmunity [114]. Th1-like Treg production is supported by IL-12 and IFN-γ. Th1-like Treg also express high levels of CXCR3 similar to Th1 cells, their effector counterpart [115].

These studies demonstrate the therapeutic potential of cytokine-activated Treg in immune diseases and transplant tolerance induction.

Th1-like Treg are decreased in the peripheral blood of patients with type I diabetes (T1D) compared to healthy controls [116]. In rheumatoid arthritis (RA), Th1-like Treg increase but these Treg have a reduced ability to suppress [117]. In non-obese diabetic (NOD) mice, ICOS^+^T-bet^+^CXCR3^+^ Treg are generated in response to increased IFN-γ production in the pancreas. ICOS^+^ Treg retain their suppressive ability as compared to ICOS^−^ Treg and delay the onset of T1D [118].

In cancer, the CXCR3^+^T-bet^+^ Treg proportion is increased in the tumour compared to lymphoid tissues, while the overall Treg proportions are not different [119]. These Th1-like Treg suppress the action of CD8^+^ T cells, allowing tumour growth [120]. Th1 cells can differentiate into Th1-like Treg in response to TGF-β signals in tumours. These suppressive Th1-like Treg not only express high levels of T-bet, but also CD39, a marker of activated Treg [121].

The pro-inflammatory effects of Th1-like Treg are also reported. In a mouse model, colitis is induced only in the presence of T-bet expressing Th1-like Treg, producing IFN-γ, that support the Th1-mediated inflammation [122].

Treg with increased IL-17 and IFN-γ production, but impaired suppressive function, are also found in patients post-liver transplantation with de novo autoimmune hepatitis. Monocytes from these patients have an upregulated production of the pro-inflammatory IL-12 and IL-6, which are most likely contributing to the loss of suppressive action by Treg [123].

### 5.1. Type 1 Cytokines

**Interferon gamma (IFN-γ).** IFN-γ is produced by both innate immune cells, such as ILC and NK cells, and adaptive immune cells like CD8^+^ T cells and Th1 cells. Signalling is mediated when IFN-γ binds to its receptor, IFNGR, which is expressed on nearly all cells. In humans, there are two forms of IFNGR: IFNGR1 and IFNGR2.

The initial IFN-γ production is promoted by IL-12 or IL-1, or upon the activation of pattern recognition receptors by tissue injury or microbial infection. Sustaining IFN-γ induction requires the activation of adaptive immune cells via the TCR recognition of antigens. IFN-γ plays an important role in dendritic cell and macrophage differentiation. The excessive generation of IFN-γ can hinder tissue repair due to the increased macrophage activation. Treg regulate IFN-γ production by tissue [124]. Promoting the activation of Treg, IFN-γ can thus promote Th1 responses or inhibit them [125].

IFN-γ can be protective and promote the resolution of inflammation in autoimmunity [126]. When produced by activated Treg, IFN-γ can bind to either IFNGR1 and IFNGR2 on effector T cells and inhibit them [127]. In addition, IFN-γ can induce activated Treg including Th1-like Treg [128].

IFN-γ^+^ Treg represent a subset of Treg induced by contact with alloantigens. A study comparing renal transplant patients with good graft function (serum creatinine of ≤1.8 mg/dL more than 100 days post-transplantation) and patients with impaired graft function (serum creatinine of ≥2.0 mg/dL) finds the former group has more CD4^+^CD25^+^Foxp3^+^IFN-γ^+^ T cells in the peripheral blood than the latter [129]. Another study reports that IFN-γ^+^ Treg, induced by IFN-γ, inhibit alloresponses non-specifically [130]. In a skin graft model, using IFN-γ-deficient mice, there is significantly impaired production and function of alloantigen-reactive Treg [37].

IFN-γ produced by Treg may influence antigen presentation through the induction of inducible nitric oxide synthase (iNOS) [109,127]. The increased production of nitric oxide by macrophages, in response to IFN-γ, may prevent immune responses against allografts by triggering apoptosis in T cells [109,127].

**Interleukin-12 (IL-12).** IL-12 was first described in 1989 as a natural killer cell stimulatory factor (NKSF) found in the supernatant of Epstein–Barr virus (EBV)-transformed B-cell lines [131]. This cytokine is encoded by two genes p40 (IL-12β) and p35 (IL-12α) that covalently bind to form bioactive heterodimer IL-12 (IL-12p70), or a bio-inactive homodimer of p40 [132]. The IL-12 receptor has two subunits: IL-12Rβ1 and IL-12Rβ2. The co-expression of both receptor IL-12Rβ1 and IL-12Rβ2 is required for the high-affinity binding to IL-12, and responsiveness to IL-12 [133].

Contrary to IL-12’s role in effector-T-cell function, IL-12 therapy prevents allograft rejection in a rat model of neonatal heart allograft rejection. This effect is mediated by IFN-γ and nitric oxide [109,110]. Further, alloantigen-specific Ts1 Treg generated by culturing naïve CD4^+^CD25^+^ Treg with IL-2 and alloantigen for four days express *Il12rb2*. The culture of Ts1 cells with rIL-12p70 (IL-12) and the same alloantigen induces Th1-like Treg. Th1-like Treg are able to suppress the proliferation of CD4^+^ effector T cells at a ratio of <1:1024 and delay cardiac allograft rejection in immunocompetent rats without any immunosuppression [54]. This is a considerably stronger effect than Ts1 cells that are produced by the activation with only IL-2 and alloantigens and suppressed at a ratio of 1:32–64 to naïve CD4^+^ T cells, or freshly isolated Treg that can only suppress T effector cells at a ratio of 1:1 [111].

Kobayashi et al. demonstrated that NKSF induces IFN-γ production, in synergy with rIL-2 [131]. IL-12 induces IFN-γ production by Th, NK, and CD8 cells [134,135], and is produced in response to bacterial stimuli or viral infection [136]. Furthermore, IL-12 inhibits IgE synthesis by IL-4-stimulated PBMCs [137].

IL-12 was initially believed to play a central role in autoimmunity until it was shown that IL-23 (a member of the IL-12 family) is the vital cytokine for orchestrating T cells and macrophages in the central nervous system [138]. Both IL-12 and IL-23 share the subunit IL-12Rβ1. Castano et al. showed that IL-12Rβ1 is not expressed by human naïve T cells or Treg, unless these cells are activated by anti-CD3/CD28 beads, which renders them more responsive to IL-12, especially the naive CD4^+^ T cells. The group also found that a high concentration of IL-12 (50 ng/mL) in culture reduced regulatory function [139].

IL-12 reduces IL-2R expression by Treg in mice, as opposed to non-Treg cells. It also decreases the Foxp3 expression and overall Treg frequency [140]. IL-12’s ability to support the activation and proliferation of effector T cells is independent of Treg [141]. IL-12’s induction of IFN-γ production by different cells, including Treg, may represent an early marker of transition of Treg into Th1 effector cells, which could be due to the loss of their suppressive function [142].

IFN-γ production by Treg in response to IL-12 is only observed in Treg expressing the transcription factor T-bet, despite the presence of high concentrations of IL-12 [143].

In a colitis model, IL-12 generates IFN-γ^+^Foxp3^+^ Treg that also produce IFN-γ and retain its suppressive function [142]. Another study found no difference in the suppressive ability of IFN-γ^+^ or IFN-γ^−^ Treg [140]. In MOG-induced EAE in mice, the administration of IL-12 shortly after induction suppresses disease and reduces the proliferation of MOG-specific T cells [144].

**Interleukin-27 (IL-27).** The IL-27 heterodimer consists of two protein subunits, p28 and EBV-induced gene 3 (EBI3). IL-27 is a member of the IL-12 family and is responsible for early Th1 differentiation until adequate IL-12 is generated [145]. IFN-β can promote IL-27 expression and inhibit IL-12 induction.

Interleukin-27 receptor subunit alpha (IL-27Rα) WSX-1 (also known as TCCR) is a cell-surface protein receptor specific for IL-27 [146]. IL-27Rα is a member of the class I cytokine receptor family and has a structural homology with the IL-6/IL-12 family of receptors, such as IL-12Rβ1 and IL-12Rβ2. IL27Rα forms a heterodimer with the common Gp130 chain [146]. Gp130 is a signal-transducing receptor used by many cytokine receptors. IL-27 signalling is involved in various immune cells, playing a crucial role in regulating-T-helper-cell differentiation and innate immunity. Mice with a TCCR deficiency present with dysfunctional Th1 responses marked by reduced IFN-γ production, which leads to increased susceptibility to intracellular infections [147].

IL-27 is produced by APC such as dendritic cells and macrophages. APC, once activated by antigens, initiate the clonal expansion of antigen-specific naïve mouse and human Th cells. IL-27 stimulates the proliferation of naïve but not memory CD4^+^ cells and, in synergy with IL-12, supports IFN-γ production by T cells and NK cells. Inducing STAT1 and T-bet, IL-27 promotes responsiveness to IL-12 and the development of Th1 cell [148]. IL-27 enhances the functions of Th1 and CD8^+^ T cells. It also promotes the development of Tfh and Tr1, and suppresses the functions of Th2, Treg, Th9, and Th17 cells [149,150,151,152,153]. In addition, IL-27 functions to directly regulate the immune activity of B cells, NK, DCs, and macrophages [154]. IL-27 can modify the effector function of CD4^+^ and CD8^+^ cells by inducing IL-10 and promoting Treg [152].

Treg express IL-27Rα. In EAE, IL-27 contributes to Treg function. Mice with Treg-specific *Il27ra*-l- develop more severe EAE [155]. IL-27 gene therapy prevents EAE development [156].

The precise effect of IL-27 on Treg is unclear although IL-27 is associated with the induction of IL-10 secretion by both T and B cells. Il-27rα^−/−^ mice have prolonged immune inflammation with infections, as CD8^+^and CD4^+^ T-cell responses persist and these mice develop a lethal CD4^+^ T-cell immune pathology with a high IFN-γ production [157]. The effect of IL-27 on IL-27Rα is to induce IL-10 expression by T cells, and promote Th1-like Treg that express Foxp3 and T-bet, as well as CXCR3. These Treg can limit Th1 responses [150]. Gut Treg release IL-27 under inflammatory conditions and this controls Th17 responses [158].

IL-27 induces CXCR3- and T-bet-expressing Treg, that differ from those induced by IFN-γ [128]. Unlike IL-12, IL-27 does not downregulate Foxp3 expression [159,160].

In a xenogeneic (human to mouse) GvHD model, IL-27-pre-stimulated human-induced Treg (iTreg) are superior in protecting recipients from GvHD [161].

**Tumour necrosis factor alpha (TNF-α).** Although TNF-α is widely seen as a pro-inflammatory cytokine, inducing apoptosis in infected cells, it also plays an important role in promoting Treg-suppressive function. TNF-α is produced by effector CD4^+^ or CD8^+^ T cells, as well as innate cells, but also produced by Treg. This cytokine can present in a soluble or transmembrane form. Treg produce membrane-bound TNF-α [162].

TNF-α binds to the TNF receptor 1 (TNFR1), which is expressed on nearly all cells, and rapidly mediates its protective function against pathogen-invaded cells. A second receptor, TNFR2, is expressed on immune cells and some non-immune cells such as endothelial cells and cancer cells. TNFR1 has a death domain in its cytoplasmic tail, whereas TNFR2 promotes survival and proliferation [163]. The transmembrane form of TNF-α preferentially binds TNFR2 [164].

TNF-α modulates immune inflammation and helps terminate the immune response. Treg that develop in thymus express TNFR2, whereas CD4^+^Foxp3^−^ cells do not express TNFR2 [165]. Only in conjunction with IL-2 is TNF-α able to activate Treg, induce their proliferation, and increase its Foxp3 expression and suppressive ability [166]. TNF-α and IL-2 can selectively activate Treg, increasing STAT5 phosphorylation. TNFR2 is highly expressed on Treg in tumour environments [167]. TNF-α utilises the NF-kB and mitogen-activated protein kinase (MAPK) pathways to exert its immunoregulatory function [168]. Additionally, TNF-α increases the expression of high-affinity IL-2 receptors, which, consequently, increases T-cell activation [169].

Treatment with TNF-α reduces autoimmune disease in mice [170,171]. TNF-α knockout mice have a delayed autoimmunity but this is prolonged and slow to recover [172].

TNF-α-rich serum provided as irradiated blood from mice with acute GvHD maintains CD25 and Foxp3 expression as well as the suppressive function of the Treg from a healthy allogeneic mouse. However, this effect is lost in the absence of the serum [173]. These TNF-α-primed Treg also improve survival and Treg function when given to mice with GvHD [173].

TNF-α also has a regulatory function in innate immunity. Mice lacking TNF-α have an exacerbated inflammatory response to lung infection including the proliferation of activated CD4^+^ and CD8^+^ T cells, the excessive production of IFN-γ and IL-12, and severe tissue damage. TNF-α gene therapy, introduced early, suppresses the proliferation of antigen-specific T cells and improves survival [174].

The pro-inflammatory effect of TNF-α is demonstrated in an in vitro study using blood from RA patients, where Treg suppress the proliferation of effector T cells, but not the production of pro-inflammatory cytokines. The TNF-α inhibitor, Infliximab, is effective in the treatment of RA, Crohn’s disease, and ulcerative colitis, with an increased frequency of functional Treg in the peripheral blood, and accompanied by attenuated inflammation [175]. In MS patients, treatment with anti-TNF-α mAb almost always leads to the exacerbation of disease [176]. RA and inflammatory bowel disease (IBD) patients benefit from anti-TNF-α treatment, but some patients develop other autoimmune diseases, including SLE and neuroinflammatory disease [177].

### 5.2. Conclusions Regarding Type 1 Responses and Treg Activation

The pathways for the activation of naïve/resting Treg by Type 1 immune responses are the most characterised, with the pathways for the induction of Th1-like Treg, by IL-2 followed by either IL-12 or IFN-γ. This results in Treg that express T-bet and Foxp3, and produce IFN-γ. These Treg express CXCR3, which promotes their migration to sites of Type 1 inflammation. They also express other markers of activation, including the high expression of CD25 and Foxp3, as well as potential suppressor molecules, such as CD39. Other Type 1 cytokines can promote Treg, including TNF-α and IL-27. Whether there is synergy between these cytokines remains to be explored. However, it appears that the persistence of IL-2 prevents the full activation of Treg in some circumstances.

## 6. Type 2 Immune Responses: Effect of Th2 Cytokines on Treg Function

**Ts2 Treg.** The second pathway of activation we demonstrated is the culture of naïve/resting Treg in the presence of IL-4 and antigens. Naïve/resting CD4^+^CD25^+^ Treg from DA rat cultured for four days with PVG alloantigens in the presence of rIL-4 are activated to Ts2 Treg, expressing Il-5rα, the receptor for late Th2 cytokine IL-5, but not *Ifngr* [43]. Ts2 Treg suppress naïve CD4^+^ T cells in MLC at a ratio of 1:32 to 1:64 (Ts2: effector T cells). Naïve/resting Treg suppress at a ratio closer to 1:1 or 1:2 [43,57]. That is a marked increase in suppression after 3–4 days of culture.

In vivo, Ts2 Treg inhibit the rejection of the specific donor PVG but not the third-party Lewis cardiac allograft, at a ratio of 1:10 to naïve CD4^+^ T cells, demonstrating their antigen specificity and enhanced suppressive ability [43], compared to naïve Treg that suppress at 1:1 [111]. Animals treated with Ts2 Treg tolerate their graft for >250 days and have similar proportions of CD4^+^CD25^+^ cells as naïve animals. CD4^+^CD25^+^ cells from these animals with surviving grafts express a higher *Il5rα* mRNA and have a low expression of IL-5, a phenotype similar to Ts2 cells. These cells have a significantly higher proliferation to specific donors, but not to third-party donors, in the presence of rIL-5, and this proliferation is blocked by anti-IL-5 antibodies [43].

The cytokine dependence of Ts2 cells is demonstrated in our studies in autoimmunity and transplantation models, where the administered IL-5 promotes Ts2 cells to suppress EAN [106] and allograft rejection [108]. The immune protective effects of parasites are shown to be mediated by IL-5, which induces more IL-5Rα-expressing Treg [178]. The immune response to parasites is a Th2 response with the production of IL-3, IL-5, and IL-13.

**Th2-like Treg.** The further activation of Ts2 cells with antigens and rIL-5 leads to the generation of Treg with a phenotypic profile similar to that of Th2 cells (Th2-like Treg), including the expression of IL-4 [179,180], IL-5, and their corresponding receptors. Th2-like Treg also express the Th2 transcription factor GATA3 [179,181] and IRF4 [45,179] in addition to STAT6 signalling [182]. Furthermore, they express other Th2 cytokines including IL-9 and IL-13. Th2-like Treg are activated Treg, being CD45RA^−^Foxp3^hi^CD25^hi^ [46,179]. This Treg subset expresses CCR4 and CCR8 but has a low CCR6 and CXCR3 expression [179].

Several studies indicate a potential role for Th2-like Treg in therapy for immune-mediated diseases. Th2-like Treg are described and characterised in cancer, especially melanoma and colorectal cancer [179], and in other conditions like arteritis, atopic dermatitis, and asthma [180,183,184].

### 6.1. Type 2 Cytokines

**Interleukin-4 (IL-4).** Discovered in 1982, IL-4 is a glycoprotein produced by mast cells, basophils, eosinophils, and activated T cells [185]. IL-4 is involved in the orchestration of the innate and adaptive immune system. Antibody production by B cells is promoted by IL-4 and so is T cell differentiation, primarily towards the Th2 lineage, a process initiated by IL-2. IL-4 is structurally homologous to IL-13, with a shared IL-4Rα, but shares only 25% of its amino acid sequences [186,187].

IL-4 signalling is dependent on the binding to IL-4Rα chain, which, in turn, can dimerise either with the common gamma chain (γc) or the IL-13Rα1 chain (CD213A1) [188]. The receptor complex involving IL-4/IL-4Rα and γc is referred to as Type 1 and is expressed by haematopoietic cells. The IL-4/IL-4Rα and IL-13Rα1 receptor complex is known as Type 2 and is expressed in non-haematopoietic cells but can also be found on haematopoietic cells [189,190,191].

Upon the binding of cytokines to the corresponding receptors, a series of intracellular signalling molecules are activated, and different Jak kinases and tyrosine kinases undergo phosphorylation [192]. The cascades of phosphorylation cause the activation of phosphatidyl-inositol 3′-kinase (PI 3-K) as well as STAT6 [193] and its nuclear localisation. Transcriptional effects promote Th2 cell generation and B-cell immunoglobulin class switching to IgE [194]. IL-4 upregulates the expression of GATA3, directly favouring a Th2 response over a Th1 response. In addition, IL-4 downregulates the Th1 IgG subclasses of autoantibodies in the serum, such as IgG3 [195,196]. The production of autoantibodies is, in turn, reduced by IL-4 neutralisation [197,198].

In patients with Takayasu’s arteritis, which involves the vasculitis of the aorta and associated major branches, Th2-like Tregs, but not Th1-like and Th17-like Treg, decrease in the peripheral blood compared to healthy individuals [180]. This is accompanied by increased levels of IL-4 and IL-13 as well as higher levels of IL-6, TNF-α, IFN-γ, and IL-17 [180]. The peripheral blood of RA patients shows higher absolute counts of Th2-like Tregs compared to healthy individuals [117]. In malignant tissue in colorectal cancer and melanoma patients, Th2-like Treg with limited suppressive function are increased compared to healthy tissue, potentially allowing cancer to thrive [179].

In liver transplant studies, treating the donor rat with IL-4 for the first five days supports the acceptance of the allografts, most likely attributed to the increased influx of immune cells including NK cells expressing IFN-γ and IDO (an enzyme involved in immune regulation) to the donor liver [199]. In contrast, treating the recipient rats with IL-4 from day 3 to day 7 post-transplantation of the liver reduces survival from >100 days (untreated rats) to 9 days with an increase in monocyte and macrophage infiltration as well as increased MHC II expression [200].

Rats receiving neonatal heart allografts treated with a combination of rIL-4 and a suboptimal dose of non-depleting anti-CD4 mAb post-transplantation have a longer survival of the transplant compared to rats using either of the treatments alone or no treatment [201]. OT to a heterotopic cardiac allograft that was induced by treatment with a non-depleting anti-CD4 mAb therapy is associated with the induction of Th1 cytokines IL-2, IFN-γ, and TNF-β, and no Th2 cytokine induction [202]. Blocking IL-4, by the administration of an anti-IL-4 mAb, does not prevent the acceptance of a specific donor-strain skin graft by OT hosts. It also does not prevent the transfer of OT to a specific donor cardiac allograft by CD4^+^ T cells [203]. Thus, in some models, IL-4 is not necessary for the induction or maintenance of OT.

In EAE, the combined treatment of rIL-4 and non-activating anti-CD3 mAb reduces the severity of active disease, associated with reduced immune cell infiltration into the brain stem compared to the treatment with non-activating anti-CD3 mAb alone [204]. This anti-CD3 mAb inhibits Th1 responses and spares Th2 induction.

IL-4’s ability to support B-cell survival, by preventing apoptosis [205], indicates IL-4 may promote autoantibody-mediated diseases. On the other hand, no role of IL-4 is demonstrated in the development of autoantibodies during the progression of SLE [206]. Others have demonstrated IL-4’s potential role as an inhibitor of autoimmunity [196,207,208].

**Interleukin-5 (IL-5).** IL-5, first known as T-cell-replacing factor (TRF), belongs to a family of cytokines including IL-3 and GM-CSF. IL-5 is produced by T cells [209,210], mast cells [211,212], innate lymphoid cells [213], and eosinophils. All three cytokines in this family share the common β receptor chain, which combines with a cytokine-specific α receptor chain. The specific receptor for IL-5 is IL-5Rα (CD125). IL-5Rα was thought to be restricted in its expression to eosinophils, mast cells, basophils, and some B cells. IL-5Rα was not believed to be expressed by T cells, until it was identified on some CD4^+^CD25^+^Foxp3^+^ Treg. We have shown that human and rodent Treg can express IL-5Rα, particularly if activated by an antigen and IL-4 [43,106].

IL-5 stimulates the production of basophils and eosinophils in bone marrow [214]. Additionally, IL-5 induces antibody secretion and class switching in B cells and affects allergic diseases in humans via B cells [215]. In mice, IL-5 increases IgE, which is produced in response to allergens. IL-5 has been described to increase the expression of the IL-2 receptor on already antigen-activated T cells, supporting their differentiation into cytotoxic T cells [216].

IL-5 therapy delays fully allogeneic neonatal heart allograft rejection and inhibits Th1 cell activation [217]. Neonatal cardiac allograft survival is significantly prolonged when rIL-5 is given in combination with anti-CD4 mAb treatment [217]. Blocking IL-5 inhibits the enhancement of graft survival, indicating this effect is IL-5-dependent. An induction of Th2 cytokine production is observed, such as IL-4 and IL-5 [217]. Th1 cytokines such as IL-2 and IFN-γ are inhibited.

It was believed that the IL-5 specific receptor IL-5Rα was not expressed by T cells. Our studies described in the previous section show CD4^+^CD25^+^ cells from DA rats cultured with rIL-4 and PVG antigens acquire IL-5Rα expression and become Ts2 cells [43], which are antigen-specific and have an enhanced ability to suppress.

Ts2 cells prevent cardiac allograft rejection for >250 days. CD4^+^CD25^+^ cells from these graft-bearing hosts also express *Il5rα* mRNA and IL-5 enhances their in vitro proliferation only with specific antigens, but not third-party antigens [43]. Moreover, rat IL-5 [218] supports their survival and antigen-specific proliferation [43].

The Ts2 and IL-5 effect is not strain-specific. rIL-5 treatment prolongs the Lewis cardiac allograft in F344 hosts, a model with one class I MHC incompatibility and no MHC II incompatibility [108]. CD4^+^CD25^+^ cells from these animals have a Ts2 phenotype expressing IL-5Rα [108]. They do not respond in vitro to specific donors, but respond to third-party antigens. IL-5 enhances these Treg’s proliferation to specific donors, but not to third-party [108].

In an adult fully-MHC-incompatible allograft model, in a high responder strain, where therapy with an anti-CD3 mAb delays rejection, the addition of rIL-5 therapy promoted long-term survival (unpublished data).

In a fully MHC mismatch and multiple minor mismatch cardiac allograft model of DA rats with a PVG graft where tolerance is induced with either cyclosporin or anti-CD4 mAb, IL-5 has shown the same effect. CD4^+^CD25^+^ cells from these animals express IL-5Rα. This IL-5Rα expression is lost upon culture with IL-4 but is retained in cultures supplemented with donor antigens and IL-5. Further, IL-5 significantly enhances donor-specific proliferation in vitro [56]. Freshly isolated CD4^+^CD25^+^ cells from these tolerant animals transfer donor-specific tolerance, and their capacity to transfer tolerance is preserved after a culture with specific alloantigens and IL-5, but not with IL-4 [56].

Another study inducing tolerance with anti-CD3 mAb found the host to have CD4^+^CD25^+^ cells, which demonstrates significantly enhanced donor-specific proliferation with IL-5 as opposed to naïve CD4^+^CD25^+^ cells [58]. These studies indicate that Treg activated to control transplant rejection express IL-5Rα, and IL-5 promotes their survival and function.

We also studied whether rIL-5 could inhibit autoimmunity. Treatment with rIL-5 reduces the severity of EAN in Lewis rats [106]. This clinical improvement is associated with the expansion of CD4^+^CD25^+^ Treg that express IL-5Rα (Ts2 cells) and proliferate to specific autoantigens, and this proliferation is enhanced by rIL-5. Blocking IL-4 inhibits the beneficial effects of rIL-5 on EAN consistent with the need for IL-4 to induce Treg to Ts2 cells that can become Th2-like Tregs [106]. Our studies in the EAE model suggests that both Ts2 cells and Th2-like Treg generated by culture of Ts2 cells with rIL-5 and specific autoantigens reduced the clinical severity of EAE (unpublished data).

The IL-5 activation of eosinophils is characteristic of a Th2 response, associated with parasite infestations. During a parasitic infestation, immune responses are impaired, in particular, autoimmunity. Parasite-infested hosts develop less severe EAE with less paralysis and weight loss compared to controls. CD4^+^CD25^+^Foxp3^+^ T cells from parasite-infested hosts express higher levels of IL-5Rα mRNA. Blocking IL-5 with an anti-IL-5 mAb abolishes the effect of parasite infestation, as does the depletion of CD25^+^ cells. Blocking IL-4 with an anti-IL-5 mAb has no effect [178,219].

As eosinophils and IgE, both are prominently present during an allergic reaction, anti-IL-5 antibodies (reslizumab and mepolizumab) have been trialled in severe asthma [220,221] with mixed results. These therapies are useful for asthma patients with eosinophilia.

**Interleukin-13 (IL-13).** Identified in 1989, IL-13 consists of 132 amino acids [222,223]. Sharing structural and functional properties with IL-4 [224], IL-13 exerts anti-inflammatory effects. IL-13 is produced by various cell types including monocytes, macrophages, mast cells, eosinophils, basophils, endothelial cells, keratinocytes, B cells, dendritic cells, and activated T cells.

The IL-13R complex consists of the subunits IL-4Rα, IL-13Rα1, and IL-13Rα2. IL-13Rα2 is mainly found on tumour cells. IL-4Rα and IL-13Rα1 are expressed by normal cells. IL-13 receptors are normally expressed on the surface of the cells that produce them, except for the T cells, where IL-13Rα1 is expressed intracellularly [191]. IL-13Rα1 alone binds to IL-13 with low affinity but forms high-affinity receptors when combined with IL-4Rα. The IL-13Rα1/IL-4Rα complex also acts as a secondary receptor to IL-4 [225]. IL-4Rα is responsible for mediating signalling. IL-13Rα2 only binds to IL-13. This binding does not result in signalling, however [226]; rather, it may induce other signalling pathways [227]. IL-13 functions via the JAK/STAT pathway.

Like IL-4, IL-13 is regularly associated with allergic immune responses, as it can induce IgG class switching in B cells [228]. IL-13 promotes tissue remodelling carried out by the structural cells like endothelial cells and keratinocytes, which express the IL-13R [229,230]. In addition, IL-13 can also stimulate the proliferation of mucus-producing goblets as well as regulate barrier function, further contributing to asthma progression [231].

IL-13 and IL-4 regulate EAE, and can endogenously maintain tolerance via Treg. Mice lacking the IL-4Rα/IL-13Rα1 heterodimer present with a reduced ability to convert their Th17 cells into Th1 cells and a dampened sensitivity to the Treg-suppressive effect. These mice are more prone to EAE, with an increased disease severity [207]. IL-13 hinders the release of cytokines like IL-17A by Th17 cells, while also preventing the development of Th17 cells.

In a setting of acute lung injury, which is often fatal due to uncontrolled local inflammation, IL-13 can have a protective role. The regulation of this inflammatory response is mediated via IL-13 production by IL-33R^+^ (ST2^+^) Treg resulting from early treatment with IL-33 [232].

IL-13 has an anti-inflammatory impact in inflammatory arthritis. Administering IL-13 and IL-4 in animals with RA results in the downregulation of pro-inflammatory cytokines, such as TNF-α, in conjunction with diminished joint inflammation and an overall improved disease prognosis [219,233,234]. An in vitro study using the peripheral blood of patients with chronic inflammatory arthritis shows reduced TNF-α and IL-1β production by macrophages in the presence of IL-13 [235]. The polymorphism of IL-13 is associated with the quicker progression of RA [236].

Our study shows rat IL-13 reduces Th1 responses [237]. DA rats receiving an allogeneic cardiac transplant from a PVG rat and treated with rIL-13 for the first 10 days post-transplantation have prolonged graft survival. The delay in graft rejection is accompanied by decreased IL-12p35, IL-12p40, and TNF-α mRNA levels in the graft, which probably cause a lower macrophage activation, and, hence, the delay in rejection [238].

**Interleukin-33 (IL-33).** IL-33 is a member of the IL-1 cytokine family and was discovered in 2005 [239]. It is expressed constitutively by stromal cells, like endothelial and epithelial cells, as well as APC and fibroblasts. This cytokine is an alarmin that responds to epithelial/endothelial damage or inflammation. IL-33 binds to the ST2 receptor, expressed on Th2 cells and innate immune cells. Upregulated in a pro-inflammatory environment, IL-33 drives the increased production of Th2 cytokines, like IL-5 and IL-13 by Th2 cells or Treg. IL-33 is involved in Th2-associated conditions, including asthma and atopic dermatitis [240,241].

Treg sorted from T1D patients’ peripheral blood have an increased ability to suppress IFN-γ production from effector T cells when cultured with IL-33 [242]. Th1 cells generate less IFN-γ, when incubated with IL-33.

Since IFN-γ is characteristically known to drive allograft rejection, treatment with IL-33 post-transplantation was examined in a chronic murine heart rejection model [243]. IL-33 prolongs allograft survival to >50 days compared to untreated controls, which have a median survival of 21.5 days. IFN-γ production does not differ between IL-33-treated ones and controls. Th2 cytokine production of IL-5, IL-13, and IL-10 is significantly increased by the cells infiltrating the graft. The generation of CD4^+^Foxp3^+^ Treg is also upregulated in the spleen of the IL-33-treated mice. In another murine cardiac transplant model, IL-33 treatment prolongs graft survival and regulates macrophage infiltration to the graft [244].

How IL-33 drives Treg expansion is explained in a murine study of CD11c^+^ dendritic cells, which secrete IL-2 upon stimulation with IL-33, selectively expanding suppressive ST2^+^ Treg [245]. IL-33 treatment specifically increases IL-2Rα gene expression [246]. ST2^+^ Treg express high levels of CD44 and ICOS, which direct the migratory and suppressive capabilities of these Treg [245,246].

In a mice model of SLE, MRL/lpr, animals treated with anti-IL-33 show decreased mortality, a higher number of Treg, and lower levels of Th17 cells [247].

### 6.2. Conclusions Regarding Type 2 Responses and Treg Activation

The pathways for the activation of CD4^+^CD25^+^Foxp3^+^ Treg during a Type 2 immune response are less well-defined. IL-4 is probably critical in the activation of Treg, and other cytokines produced late during the Th2 response, such as IL-5 and IL-33, play a major role in amplifying the effect. Many of these cytokines such as IL-13 have a direct anti-inflammatory effect, which may mask any effect they have on Treg. The presence of Th2-like Treg in malignant tissues may contribute to the inhibition of effector immune responses and needs further study.

## 7. Type 3 Immune Responses

**Th17 cells.** Naïve T cells differentiate into Th17 cells in response to TGF-β and IL-6. Th17 cells produce IL-17A and IL-17E [248,249]. The Th17 response is involved in the tissue destruction and inflammation seen in many autoimmune diseases including MS, rheumatoid arthritis, psoriasis, Crohn’s disease, and ulcerative colitis [250].

IL-22 and IL-23 also promote Th17 cells. IL-23 maintains the commitment to the Th17 lineage [251,252]. IL-22 production by Th17 cells is induced by IL-6 and blocked by TGF-β. IL-23 is produced by activated T cells or monocytes [253].

IL-27 has also been found to inhibit the development of Th17 cells and is a potential therapeutic target for Th17-driven autoimmune diseases [254].

While Th17 cells are known to be promoters of inflammation, Th17-like Treg have the potential to control inflammatory responses.

**Th17-like Treg.** Th17-like Treg are a subset of CD4^+^Foxp3^+^ Treg that express CCR6 and the transcription factor retinoic-acid-related orphan receptor γt (RORγt) while producing IL-17. Th17-like Treg are similar to Th17 cells [249,255] except they also express Foxp3. Both Th17 and Th17-like Treg express the transcription factor STAT4. Th17-like Treg produce IL-17, IL-10, and IL-22. Th17-like Treg suppress the proliferation of CD4^+^ T cells [42]. IL-17-producing Treg may differentiate in the periphery [42] and are not found in the thymus.

CD4^+^Foxp3^+^CCR6^−^ Treg differentiate into IL-17-producing Treg in response to antigen stimulation and specific cytokines such as IL-1β, IL-2, IL-23, and IL-21. CCR6 expression directs the migration of both Th17 and Th17-like Treg to sites of inflammation in tissues [256].

Th17-like Treg are found in patients with psoriasis [257] and active SLE [258]. IL-6 induces Treg into Th17-like Treg in the active SLE patients. Th17-like Treg have similar phenotypic and functional characteristics to their effector counterpart Th17 cells [258]. Notably, the Foxp3 expression in IL-17-producing Treg is often transient, suggesting that Th17-like Tregs may represent an intermediate population capable of progressing toward a Th17 effector phenotype [259,260]. Th17-like Treg remain suppressive until they are induced to produce IL-17 in the presence of pro-inflammatory cytokines such as IL-6. These findings align with the concept of Treg plasticity, where environmental cues, like cytokines, can determine the fate of a Treg.

Both conventional CD4^+^ T cells and CD4^+^Foxp3^+^ cells producing IL-17 are elevated in the peripheral blood of patients with juvenile idiopathic arthritis [261]. In contrast, the peripheral blood of T1D patients has decreased Th17-like Treg compared to healthy controls, while the proportion of effector Th17 cells is increased [116].

### Type 3 Cytokines

**Transforming growth factor beta (TGF-β).** The TGF-β superfamily includes over 30 proteins involving TGF-βs, activin, and growth differentiation factors. In mammals, TGF-β is present in three different isoforms, TGF-β1, TGF-β2, and TGF-β3. TGF-β1 is the most prominent form in the immune system [262,263,264]. Most leukocytes, including lymphocytes and platelets, produce TGF-β. TGF-β acts through a specific TGF-β receptor.

TGF-β1 is involved in regulating many cells’ functions including cell growth, proliferation, differentiation, and apoptosis. Further, TGF-β has many effects on the immune system including suppressing cytotoxic T cells, and Th1 and Th2 cells [265]. In turn, TGF-β promotes tissue repair and wound healing and inhibits tumours [266,267].

TGF-β1 inhibits the activated-T-cell proliferation induced by IL-1 or IL-2. It also inhibits other cytokines, including IFN-α, TNF-α, and other cytokines. TGF-β1 suppresses monocytes’ and macrophages’ inflammatory responses. At low levels, TGF-β can induce the monocyte expression of IL-1β and TNF-α [79].

TGF-β in conjunction with IL-6 induces Th17 cells [249]. However, TGF-β alone in response to antigens, in the absence of inflammatory cytokines such as IL-6 or IL-1, induces CD4^+^CD25^−^Foxp3^−^ T cells in the periphery toward a Treg phenotype, which is the CD4^+^CD25^+^Foxp3^+^ cell. CD4^+^CD25^+^Foxp3^+^ cells are iTreg and they can inhibit an immune response and are discussed in a separate section.

TGF-β is one of the anti-inflammatory cytokines produced by Treg CD4^+^CD25^+^Foxp3^+^ Treg in the thymus. TGF-β1 has an indispensable role in the development, function and maintenance of Treg [268,269]. This is highlighted in mice where the deletion of the TGF-β1 receptor from T cells results in a reduced frequency of thymic Treg. Treg recover in the presence of IL-2. When the IL-2 gene is ablated, Treg are generated in neither the thymus nor the periphery [270].

**Interleukin-23 (IL-23).** IL-23 is a pro-inflammatory cytokine produced by macrophages and APC. It is a member of the IL-12 cytokine family and is a heterodimer comprising two subunits IL-23A and IL-12β encoded by genes p19 and p40, respectively. IL-23 stimulates Type 3 immune responses, especially contributing to Th17 cell development [252], in the presence of TGF-β and IL-6 or IL-1 [271]. IL-23 binds to IL-23R, to support Th17 cells, but has no effect on its proliferation or the commitment of naïve T cells to the Th17 lineage [272,273]. Importantly, IL-23 promotes the Th17 cell production of pro-inflammatory cytokines such as IL-17A, IL-17F, IL-22, GMCSF, IFN-γ, and TNF-α.

IL-23R is expressed by Th17-like Treg in the colon but less in other compartments. IL-23 inhibits these IL-23R-expressing Treg, reducing their regulation of immune cells [274].

Treg expressing IL-23R are affected by IL-23 produced by tumour-infiltrating macrophages. This increases the suppressor potency of these cells, allowing an increased tumour growth. Treg that are *Il23r*^−/−^ are incapable of suppressing [275]. Blocking IL-23R impairs Treg in tumours and enhances anti-tumour immunotherapy [276].

IL-23R-expressing CAR Treg have been developed to treat Crohn’s disease, consistent with IL-23/IL-23R being a target for the treatment of autoimmune diseases [277].

**T follicular helper (Tfh) cells.** T follicular helper (Tfh) cells are a distinct subset of CD4^+^ T cells that express the transcription factor Bcl6, produce IL-21, and express the chemokine receptor CXCR5. Tfh cells are essential for germinal centre (GC) formation and the regulation of B-cell-mediated humoral immunity. By providing help to B cells in secondary lymphoid organs, Tfh cells enable the immunoglobulin class-switch recombination, and the generation of long-lived plasma and memory B cells [278]. Their function is tightly orchestrated by BCL6, the master transcription factor for the Tfh lineage [279,280]. Tfh express surface molecules (e.g., CXCR5, PD-1, and ICOS), and cytokine signals—particularly IL-21, IL-10, and IFN-γ, the signature cytokines of Tfh cells [281,282,283].

**T follicular Regulatory cells (Tfr).** T follicular Regulatory cells (Tfr) are a small proportion of the total Treg pool. They regulate interactions between B cells and Tfh cells within the germinal centre [284]. In humans, these cells are CD4^+^CD25^+^Foxp3^+^CXCR5^+^ cells. Like Tfh cells, Tfr cells express CXCR5, PD-1, ICOS, and CD44. Tfr express Bcl6, the transcription factor of Tfh cells, along with Blimp-1. They also have a Treg-like phenotype, expressing Foxp3, CD25, and CTLA4, which are not expressed by Tfh cells. Tfr numbers are regulated through a balance between Bcl-6 and Blimp-1. Despite sharing a phenotype with Tfh cells, Tfr cells do not produce IL-21 and IL-4.

**Figure 8:**
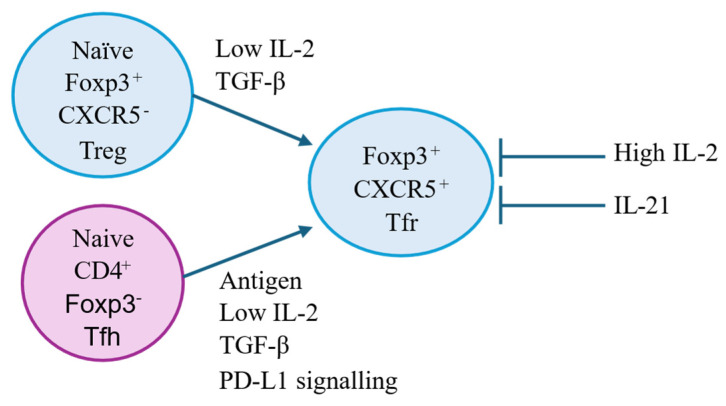
Generation of CXCR5^+^Foxp3^+^ T follicular regulatory cells (Tfr) from naïve Foxp3^+^CXCR5^−^ Treg or naïve CD4^+^Foxp3^−^ T follicular helper (Tfh) cells in presence of low IL-2 and TGF-β. Presence of IL-21 or high concentration of IL-2 inhibit Tfr generation.

The differentiation and activation of Tfr may occur through different mechanisms (Figure 8). Tfr are not thought to be produced in the thymus but may be activated in the periphery from naïve/resting Foxp3^+^CXCR5^−^ Treg by TGF-β in the presence of a low IL-2 [285]. Alternatively, these can be produced from naïve CD4^+^Foxp3^−^ Tfh cells via PD-L1 signalling, or the presence of TGF-β, or low levels of IL-2 or antigens [286].

The cytokine milieu can affect the generation of Tfr. IL-21 and high levels of IL-2 can inhibit Tfr development [287,288]. IL-10 secreted by Tfr can influence Tfr-mediated antibody suppression. In the absence of Tfr cells, the cytokine production by Tfh cells, including IL-10, IFN-γ, and IL-21, is much higher, suggesting Tfr control their production by Tfh cells [289].

Tfr cells can modulate antibody responses to alloantigens and, hence, have an important role in graft rejection [290].

## 8. CD4^+^ Treg Induced from CD4^+^CD25^−^Foxp3^−^ T Cells

In illustrations of CD4^+^ T-cell activation, often, Treg are included as an extra pathway of CD4^+^ cell activation, which occurs in the presence of TGF-β and the absence of inflammatory cytokines such as IL-6 and IL-1. This pathway occurs in the periphery, and these cells are known as peripheral Treg (pTreg) or iTreg [291]. Other subtypes of Treg are induced from CD4^+^CD25^−^Foxp3^−^ T cells due to antigen activation and different subpopulations of cytokines, including Tr1 and iTr35. However, naturally occurring thymus-derived CD4^+^CD25^+^Foxp3^+^ Treg represent the major population of regulatory cells.

**Figure 9:**
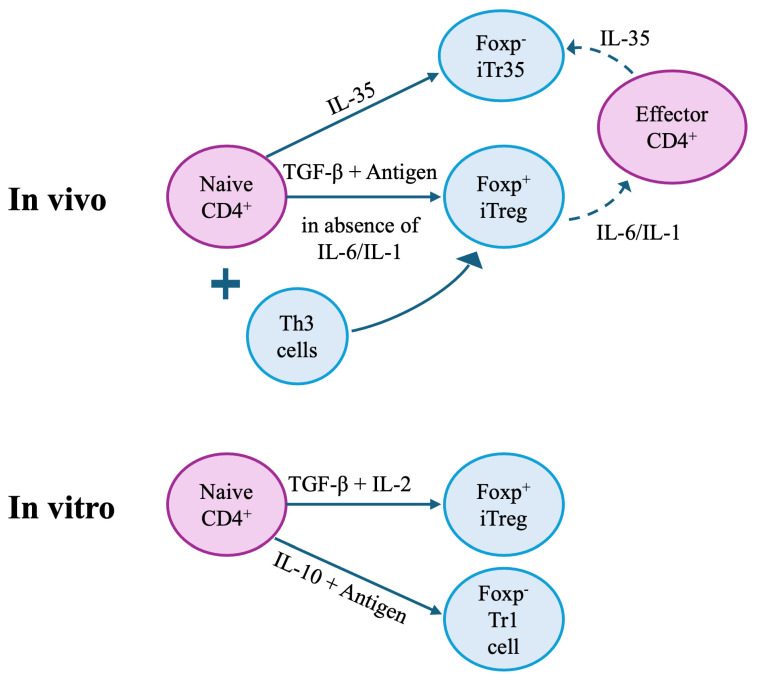
Induction of various subtypes of Treg including induced Treg (iTreg), IL-35-producing regulatory T cells (iTr35), T helper 3 cells (Th3) and Type 1 regulatory cells (Tr1) from naïve or effector T cells is dependent on the presence/absence of specific cytokines and antigen stimulation. The cell plasticity is highlighted by the dotted arrows displaying how iTreg can be converted into effector CD4^+^ cells in the presence of pro-inflammatory cytokines such as IL-6 or IL-1. These effector CD4^+^ cells can then be converted back to another type of Treg, namely iTr35 via IL-35 support.

**iTreg.** iTreg are generated in the periphery from naïve T cells in response to antigen stimulation and TGF-β in the absence of inflammatory cytokines such as IL-6 and IL-1 (Figure 9). These transiently express Foxp3, but their Foxp3 expression is not stable as they lack the Treg-specific demethylated region (TSDR). These cells can revert to effector cells if exposed to inflammatory cytokines such as IL-6 or IL-1. iTreg can also be induced in vitro by the culture of T cells with TGF-β and IL-2 (Figure 9) [292]. Many excellent reviews are available on iTreg [291].

**T helper 3 cell (Th3).** Th3 cells play a key role in peripheral immune regulation. Th3 cells were first identified in studies of oral tolerance, particularly within the gut-associated lymphoid tissue [293]. Th3 are CD4^+^ cells and secrete high levels of TGF-β. They do not express Foxp3. Th3 arise in the periphery and mediate suppression primarily through TGF-β [293]. Th3 cells are typically induced in mucosal environments following antigen exposure under non-inflammatory conditions, such as the ingestion of dietary antigens or exposure to commensal microbiota.

Much of the interest in Th3 was in the 90s; however, in recent years, limited work has been reported.

TGF-β produced by Th3 cells exerts broad regulatory effects, including the suppression of effector T-cell proliferation [294], the inhibition of Th1, Th2, and Th17 differentiation, and the promotion of the conversion of conventional T cells into Foxp3^+^ iTreg. TGF-β plays a role in maintaining the epithelial barrier integrity and inducing IgA class switching in B cells, which is critical for mucosal immunity [295,296,297].

In autoimmune diseases, Th3 cells are protective regulators. In MS, TGF-β limits central nervous system inflammation and promotes the stability of the blood–brain barrier. In EAE, enhancing TGF-β signalling or increasing Th3 cell activity ameliorates disease symptoms [298,299].

In IBD, where the immune tolerance to intestinal antigens is disrupted, Th3 cells are central to maintaining mucosal homeostasis [300]. Their presence correlates with improved gut barrier function and reduced inflammation, underscoring their importance in diseases such as Crohn’s disease and ulcerative colitis [301]. In RA, while the role of TGF-β is more complex due to its involvement in tissue remodelling and fibrosis, appropriately regulated TGF-β from Th3 cells can suppress the inflammatory T-cell response and reduce joint damage [302,303].

**Tr1 cells.** Tr1 cells are another Treg subset, but they do not express Foxp3. These cells are induced from CD4^+^CD25^−^Foxp3^−^ T cells by repeated culture with an antigen and IL-10 (Figure 9) [32]. Tr1 cells produce IFN-γ, IL-10. and IL-5, but no IL-4 and limited IL-2, and mediate suppression through the secretion of IL-10 [32].

Tr1 cells typically arise in response to persistent antigen stimulation, often under tolerogenic conditions, such as those mediated by IL-10-producing dendritic cells [304]. Tr1 cells can co-express CD49b and LAG-3 in both humans and mice [305].

IL-10 produced by Tr1 acts by inhibiting APC activation, reducing MHC class II and costimulatory molecule expression, and suppressing pro-inflammatory cytokine release including IL-12, TNF-α, and IL-6 [306]. IL-10 also directly suppresses Th1 and Th17 cell proliferation and cytokine production, mediating the Tr1 effect in controlling excessive or chronic inflammation [307].

Tr1 cells are protective in multiple autoimmune diseases [308,309,310]. Reduced IL-10-producing Tr1 is associated with disease progression [311]. Similarly, a relative deficiency of Tr1 cells is observed in T1D patients [312]. In IBD, Tr1 cells are essential for maintaining immune tolerance in the gut [313]. Patients with IBD often exhibit reduced Tr1 frequencies or impaired IL-10 secretion, contributing to uncontrolled intestinal inflammation. In an animal model of RA (collagen-induced arthritis, CIA), mice with IL-10^−/−^ B cells showed reduced Tr1 cells and increased pro-inflammatory cytokine production, such as IL-17 and IFN-γ, accompanied by worsened disease [314]. Restoring Tr1 cell numbers or IL-10 production can help control joint inflammation and limit autoimmune progression [314]. In the murine model of EAE, Tr1 cells limit encephalitogenic T-cell responses and reduce the disease severity [315]. Tr1 cells help suppress inflammatory responses to commensal bacteria and dietary antigens [316].

**Interleukin-10 (IL-10).** IL-10 is produced by different leukocytes including lymphocytes (T cells and B cells), and granulocytes, such as neutrophils and eosinophils. IL-10 can mediate its immunosuppressive function by binding to its receptor IL-10R, a hetero-tetramer consisting of two IL-10Rα (IL-10R1) and two IL-10Rβ (IL-10R2) molecules [317]. IL-10Rα is the binding subunit, while IL-10Rβ serves as the signalling subunit. However, IL-10Rα, once bound to IL-10, might induce conformational changes in IL-10Rβ, allowing it to also bind IL-10 [318]. While IL-10Rα only responds to IL-10, IL-10Rβ forms a part of receptor complexes for multiple cytokines including IL-22 [319,320], IL-26 [321], and interferon lambda (IFN-λ) [322]. Mutations in the IL-10R subunit protein have been found in IBD patients, highlighting the importance of IL-10 in maintaining immune homeostasis [323,324].

IL-10 can inhibit the production of pro-inflammatory IFN-γ and other cytokines by Th1 cells [325], and has, therefore, been trialled to treat inflammatory diseases. However, higher doses of IL-10 seem to have an opposite effect, increasing the production of IFN-γ [326]. IL-10 therapy has also been halted by the poor delivery of IL-10 to the gastrointestinal tract [327,328]. Studies are underway to use modified IL-10 that can prevent monocyte/macrophage activation while not inducing IFN-γ production [329,330].

**iTr35.** iTr35 are a type of iTreg that are generated by the action of IL-35 on effector T cells in the periphery, converting them to IL-35-producing Treg (iTr35) [48]. iTr35 cells do not express Foxp3 and are highly suppressive. These mediate regulation by the secretion of IL-35, which controls the differentiation and proliferation of effector T cells. The role of iTr35 has been implicated in the control of various autoimmune diseases, allergy, and infection.

**Interleukin-35 (IL-35).** IL-35 was identified in early 2000s. It belongs to the IL-12 family together with IL-12, IL-23, and IL-27. It is a heterodimer of IL-12p35 (IL-12α chain) and EBI3 (IL-27β chain). The IL-35 subunit EBI3 is shared with IL-27 [331]. Unlike other members of the same family, IL-35 is not produced by APC. Mice Treg produce IL-35 constitutively, but human Treg only produce it upon activation. In addition to being produced by Treg, it also supports Treg and their suppressive function [332,333].

IL-35 induces peripheral iTr35, a population of Treg expressing IL-35 but lacking Foxp3 expression. IL-35 also induces IL-10-producing regulatory B cells (IL-10^+^ Breg) and IL-35-producing regulatory B cells (IL-35^+^ Breg).

The IL-35 receptor (IL-35R) is composed of three units, the IL-12Rβ2 chain, IL-27Rα chain, and gp130. The functional receptor can be a homodimer or heterodimer depending on the configuration of these three units. IL-12Rβ2 is responsible for the suppressive effect of IL-35 [334].

Not only does IL-35 support Treg function, but it can also convert conventional effector T cells into IL-35-producing Treg (iTr35 cells) that can prevent the proliferation and differentiation of effector T cells [48]. In vitro, IL-35 supports the proliferation of Treg, maintaining the Foxp3 expression and suppressive capacity while suppressing Th17 differentiation [335].

In mice, IL-35 promotes the suppressive function of Treg and inhibits collagen-induced arthritis (CIA) [335] and atherosclerosis [336,337]. The inhibition of atherosclerosis is shown to be via the CCR5-amplified Treg suppressive mechanism [337]. IL-35-supported CCR5^+^ Treg contribute via three mechanisms: increased migration, the inhibition of AKT-mTOR signalling, and promotion signalling via inhibitory checkpoint inhibitors like TIGIT and PD-1.

IL-35 has therapeutic benefits in mice models of heart allograft rejection by tipping the balance toward immune regulation [338]. IL-35 treatment delays acute rejection combined with a decreased mononuclear cell infiltration, specifically CD8^+^ T cells in graft, and reduced vasculitis and cardiomyocyte necrosis, while promoting CD4^+^Foxp3^+^ Treg. Mice depleted of Treg before transplantation do not show the beneficial effect of IL-35 treatment [338].

Together, peripherally induced Treg including iTreg, Th3, Tr1, and iTr35 are likely to be an essential part of the peripheral regulatory network that maintains immune equilibrium and prevents autoimmunity.

## 9. Concluding Remarks

Treg, like other lymphocytes and non-lymphoid cells involved in inflammation, can be induced or inhibited by many different cytokines. As part of their activation, Treg can produce cytokines that either activate or inhibit other cells. Broadly, the type of response of CD4^+^ T cell drives the activation of other cells. Thus, Th1 cytokines IFN-γ and IL-12 drive the activation of Treg to Th1-like Treg. Paradoxically, IL-2, the cytokine that drives the initial activation of Treg, inhibits the differentiation of Th1-like Treg. Th1-like Treg are the most potent suppressing Treg described, being hundred-fold more potent than resting/naïve Treg. Such potent inhibition is required to prevent allograft rejection, GvHD, and autoimmunity.

To date, much work has focused on IL-2; yet, IL-2, while promoting the initial activation of CD4^+^CD25^+^Foxp3^+^ Treg, can block the full potential inhibitory capacity of these Treg when exposed to cytokines of an ongoing immune response. This is when the production of early cytokines such as IL-2 and IL-4 wanes and the secretion of other cytokines increases.

This review attempts to summarise the effects of many cytokines, and, while the effects of individual cytokines can be isolated in many instances, the number of permutations is large. A more precise understanding of the multiple potential pathways of activation of Treg is required, so potent antigen-specific Tregs can be generated with cytokines other than IL-2.

Authors’ note: During the preparation of the manuscript, the Nobel committee awarded the Prize for Physiology or Medicine in 2025 for the work on peripheral tolerance including regulatory T cells and discovery of Foxp3 as the gene that promoted Regulatory T cells. Brunkow and Ramsdell cloned Foxp3 in scurfy mice which have spontaneous autoimmunity and showed cells with wild-type Foxp3 gene prevented the development of autoimmunity [33]. The discovery of Foxp3 identified the CD4^+^CD25^+^ T cells that prevent autoimmunity in neonatally thymectomised mice, described by Sakaguchi [31], and which mediate Transplant tolerance [8].

## Data Availability

No new data were created or analyzed in this study. Data sharing is not applicable to this article.

## Abbreviations

The following abbreviations are used in this manuscript:

APC	Antigen-presenting cells
Con A	Concanavalin A
DC	Dendritic cell
EAE	Experimental autoimmune encephalomyelitis
EAN	Experimental autoimmune neuritis
EBV	Epstein–Barr virus
GM-CSF	Granulocyte-macrophage colony-stimulating factor
GVHD	Graft-versus-host disease
ILC	Innate lymphoid cell
mAb	Monoclonal antibody
MAPK	Mitogen-activated protein kinases
MBP	Myelin basic protein
MLC	Mixed lymphocyte culture
MS	Multiple sclerosis
NK	Natural killer
PNM	Peripheral nerve myelin
R	Receptor
RA	Rheumatoid arthritis
s/n	Supernatant
SLE	Systemic lupus erythematosus
Treg	T regulatory cell
TRF	T-cell-replacing factor
T1D	Type 1 diabetes
γc	Gamma chain
